# Miscellany

**Published:** 1901-05

**Authors:** 


### Miscellany.

Oxyphosphate of Copper.—Dr. Mansell, in his report to the *Dental Record* of the proceedings of the International Dental Congress of 1900, at Paris, makes mention of a new filling-material,—Ames's oxyphosphate of copper. It consists of a liquid and powder, and is mixed and used like other cements. The powder, unfortunately, is black.

This cement is very plastic and sets quickly at the temperature of the mouth. It is especially suitable for filling temporary teeth, first permanent molars, and almost inaccessible cavities, and for setting shell crowns. Dr. Ames claims that it is not necessary to remove all the softened dentine in filling, as the oxide of copper sterilizes and hardens it. It will, if placed over an exposed pulp in a temporary tooth, prevent it from suppurating. If mixed thin, it will flow into fissures and pits and adhere firmly. The filling must be trimmed quickly, because it is very hard and difficult to cut when thoroughly set.

Dr. Ames believes it will prove of great value in setting crowns, because it will be less liable to become foul than ordinary cement. It has not been used long enough to test its lasting qualities.

---

A Durable Cement.—Dr. W. W. Flora, of Carthage, Mo., has in his possession a specimen of bridge-work that had been done by a native dentist of India fifty-three years ago, in which the dentist had used natural teeth. They were attached to the bars by gold wire running through the length of the teeth and soldered to the bars. A cement was used that had stood the test fifty-three years with perfect color, something that is a marvel to the modern manufacturer of cement.—*Western Dental Journal.*

---

A New Method of making Dies.—The *Ohio Dental Journal* gives a method of making metal dies described in "Dental Clippings" as follows: A plaster impression is taken of the mouth, but instead of waxing up the pieces, they must be set to place with cement. The impression is then boiled for ten or fifteen minutes in beeswax. While still hot the surplus wax is drained off, taking care that none of the finer lines have been filled, the idea being simply to fill the pores of the plaster. A piece of sheet wax—beeswax is best—is built up around the tray to the height of the desired die. The whole is now given a thorough coating of dry graphite, the difficult parts being reached with a fine camel's-hair pencil. This is electro-plated with copper to about the thickness of a visiting-card. Molten zinc can then be poured into the matrix thus formed. In order to avoid accidents, it is best to set the impression in a dish of sand when the zinc is run.

The counter-die can be made of any of the metals or alloys used for this purpose, as the copper fuses at a very high temperature.

This method is especially applicable where there are undercuts, doing away with "cases." It gives a die which cannot be duplicated for accuracy; and although it takes some time to describe it, the whole process takes but a very little time. It can be plated over night, and in the morning the die can be run.

